# BILINGUAL SPEAKERS POSTPONE SYMPTOMS OF COGNITIVE DEFICIT IN PARKINSON’S DISEASE

**DOI:** 10.1093/geroni/igz038.2447

**Published:** 2019-11-08

**Authors:** Ladan Ghazi Saidi

**Affiliations:** University of Nebraska at Kearney, Kearney, Nebraska, United States

## Abstract

Maintaining cognitive abilities despite healthy aging, neurodegeneration or acute damage is known as cognitive reserve (Stern, 2002; Stern, et. al., 2018). There is evidence for a higher cognitive reserve in bilingual speakers (Kavé, et. al., 2008), mainly due to their improved executive functioning and attention. Thus, I hypothesized that patients with Parkinson’s disease would manifest PD related cognitive symptoms later than monolinguals as a result of better compensation. The aim of this study is to explore how bilingualism affects cognitive abilities in PD patients with cognitive deficit. QPN publicly available database was used to analyze the data on PD patients with (PD-CD) and without (PD) cognitive deficit and their demographic information including the number of spoken languages. Monolingual PD and PD-CD patients were compared to their bi- and multilingual peers on their age of on-set of their cognitive decline as well as descriptive demographic information. The results showed that PD-CD patients who speak more than one language manifest symptoms of cognitive impairment at least three years later than their monolingual peers. These results bring evidence that life-long bilingualism contributes to a stronger cognitive reserve and better compensation in case of a neurodegenerative disorder such as PD.

